# Radiodynamic therapy with CsI(na)@MgO nanoparticles and 5-aminolevulinic acid

**DOI:** 10.1186/s12951-022-01537-z

**Published:** 2022-07-16

**Authors:** Fangchao Jiang, Chaebin Lee, Weizhong Zhang, Wen Jiang, Zhengwei Cao, Harrison Byron Chong, Wei Yang, Shuyue Zhan, Jianwen Li, Yong Teng, Zibo Li, Jin Xie

**Affiliations:** 1 Department of Chemistry, University of Georgia, Athens, GA 30602 USA; 2 Department of Hematology and Medical Oncology & Winship Cancer Institute, Emory University School of Medicine, Atlanta, GA 30322 USA; 3 Department of Radiology, University of North Carolina at Chapel Hill, Chapel Hill, NC 27599 USA

**Keywords:** Nanoparticles, Radiation therapy, Photodynamic therapy, Cancer, Scintillator

## Abstract

**Background:**

Radiodynamic therapy (RDT) holds the potential to overcome the shallow tissue penetration issue associated with conventional photodynamic therapy (PDT). To this end, complex and sometimes toxic scintillator–photosensitizer nanoconjugates are often used, posing barriers for large-scale manufacturing and regulatory approval.

**Methods:**

Herein, we report a streamlined RDT strategy based on CsI(Na)@MgO nanoparticles and 5-aminolevulinic acid (5-ALA). 5-ALA is a clinically approved photosensitizer, converted to protoporphyrin IX (PpIX) in cancer cells’ mitochondria. CsI(Na)@MgO nanoparticles produce strong ~ 410 nm X-ray luminescence, which matches the Soret band of PpIX. We hypothesize that the CsI(Na)@MgO-and-5-ALA combination can mediate RDT wherein mitochondria-targeted PDT synergizes with DNA-targeted irradiation for efficient cancer cell killing. Because scintillator nanoparticles and photosensitizer are administered separately, the approach forgoes issues such as self-quenching or uncontrolled release of photosensitizers.

**Results:**

When tested in vitro with 4T1 cells, the CsI(Na)@MgO and 5-ALA combination elevated radiation-induced reactive oxygen species (ROS), enhancing damages to mitochondria, DNA, and lipids, eventually reducing cell proliferation and clonogenicity. When tested in vivo in 4T1 models, RDT with the CsI(Na)@MgO and 5-ALA combination significantly improved tumor suppression and animal survival relative to radiation therapy (RT) alone. After treatment, the scintillator nanoparticles, made of low-toxic alkali and halide elements, were efficiently excreted, causing no detectable harm to the hosts.

**Conclusions:**

Our studies show that separately administering CsI(Na)@MgO nanoparticles and 5-ALA represents a safe and streamlined RDT approach with potential in clinical translation.

**Graphical Abstract:**

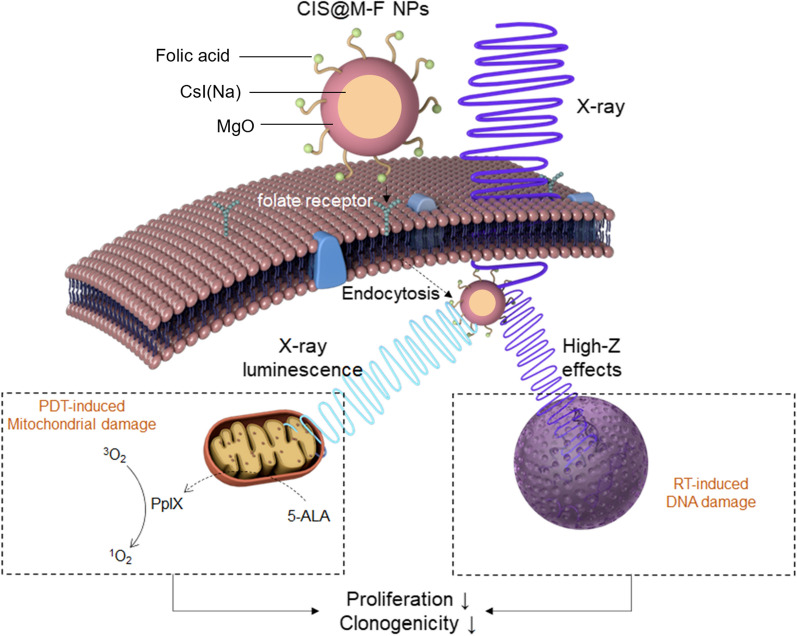

**Supplementary Information:**

The online version contains supplementary material available at 10.1186/s12951-022-01537-z.

## Background

PDT is an emerging cancer treatment modality [1, 2]. During PDT, a photosensitizer (PS, both singular and plural) [3–5] molecule is activated by light to produce ROS, most importantly singlet oxygen (^1^O_2_), which cause cancer cell death [6–8]. Light and PS need to be colocalized to mediate toxicity, rendering PDT innately selective. PDT can be delivered repeatedly without incurring resistance to therapy, and can be combined with both chemotherapies and immunotherapies for enhanced efficacy [9–12]. Despite these merits, PDT has not yet been accepted as a mainstream cancer treatment option. One major caveat is that light cannot deeply penetrate biological tissues, precluding PDT from treatment of large and/or multifocal, deep-seated tumors [13]. To address the issue, others and us have developed a modality known as radiodynamic therapy (RDT) or X-ray induced photodynamic therapy (X-PDT) [14–17]. RDT or X-PDT is often mediated with a scintillator-PS nanoconjugate [18–20], in which the scintillator down-converts X-ray photons to visible photons that activate the PS [21]. Leveraging the excellent tissue penetration of X-rays, RDT offers a solution to the restricted tissue penetration problem.

Multiple nanoplatforms have been tested for RDT [22–31]. For instance, Chen et al. demonstrated that LaF_3_:Tb, ZnS:Cu,Co, and copper-cysteamine nanoparticles can mediate RDT [24–26]. Lin et al. synthesized a series of metal-organic frameworks and confirmed their ability to enhance cancer cell killing under radiation [29–31]; one formulation, RiMO-301, is being investigated in the clinic [32]. We have prepared SrAl_2_O_4_:Eu^2+^-MC540 and LiGa_5_O_8_:Cr^3+^-2,3-naphthalocyanine nanoparticle conjugates [22, 23] and confirmed their radiosensitizing efficacy [14, 22, 23]. However, PS may experience self-quenching or pre-mature release in these nanoconjugates, limiting activation efficacy. Some scintillator nanoparticles are made from top-down approaches, which are associated with relatively large batch-to-batch variations. Furthermore, scintillators often contain toxic transition or lanthanide elements, and their long-term side effects remain to be fully investigated. Overall, these issues pose as barriers for large-scale manufacturing and regulatory approval of the technologies.

Herein we explore a streamlined RDT strategy based on CsI(Na)@MgO nanoparticles and 5-aminolevulinic acid (5-ALA). Cesium iodides such as CsI(Na) are established scintillation materials with high light outputs [e.g. 42,000 photons/MeV for CsI(Na)] [33]. The emission of CsI(Na) perfectly matches the absorbance of PpIX, a biosynthetic product of 5-ALA [34]. Owing to the Warburg effect, cancer cells downregulate ferrochelatase, an enzyme that incorporates Fe^2+^ into PpIX in the final step of heme synthesis [35], resulting in selective accumulation of PpIX in cancer cells’ mitochondria [36]. This tumor targeting ability has motivated the use of 5-ALA as either a PS [37–39] or an imaging agent [40] in the clinic. We postulate that, when irradiated, CsI(Na) nanoparticles can activate 5-ALA-induced PpIX, causing cancer cell death. Of note, clinical 5-ALA PDT employs red light to activate the 630-nm Q-band of PpIX, which has relatively low absorptivity. This compromise is necessary because shorter wavelength photons would be largely scattered by the skin or absorbed by pigments such as melanins. In the current approach, the light source, i.e. CsI(Na) nanoparticles, are delivered into cancer cells and illuminate therein, minimizing light attenuation. The ~ 410 nm luminescence from CsI(Na) activates the Soret band of PpIX, which absorbs at least one order of magnitude more strongly than the 630-nm Q-band, potentially improving efficacy. Cesium iodide is highly water soluble, so we coat CsI(Na) nanoparticles with a layer of MgO to prevent fast degradation. We also impart DSPE-PEG-Folate to nanoparticle surface to render them with good colloidal stability and tumor targeting ability.

**Scheme 1:**
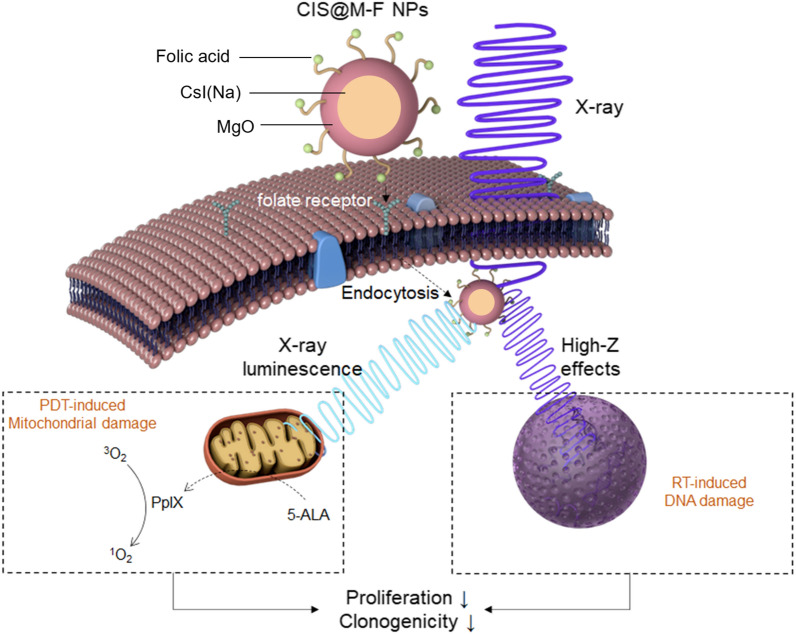
CsI(Na)@MgO@DSPE-Folate nanoparticles (CIS@M-F) and 5-ALA are separately administered but colocalized in cancer cells to enable RDT. CIS@M-F are internalized by cancer cells through receptor-mediated endocytosis. 5-ALA is converted to PpIX, which accumulates in cancer cells’ mitochondria. When irradiated, CIS@M-F produce X-ray luminescence that activates PpIX, causing mitochondria damage. Meanwhile, CIS@M-F also afford high-Z effects that enhance RT-induced DNA damage. The two components synergize to reduce cancer cell proliferation and tumorigenicity

The current RDT strategy affords several benefits. First, there is no need to load PS onto CsI(Na)@MgO nanoparticles, forgoing issues such as self-quenching among PS molecules and uncontrolled release of them. CsI(Na)@MgO nanoparticles are synthesized through wet chemistry with excellent reproducibility and scalability. Second, CsI(Na) nanoparticles comprise no transition and lanthanide elements. After treatment, the nanoparticles are degraded to low-toxic alkali and halogen ions, which are safely excreted from the hosts. Third, the PDT component specifically targets mitochondria, which are 5–10 times more susceptible to PDT than plasma membrane and endosomes/lysosomes [41, 42] that are common targets of previous RDT nanoplatforms. We expect the mitochondria-targeted component to synergize with the DNA-target RT, improving cancer cell killing (Scheme 1). We evaluated the efficacy of the approach first in vitro with 4T1 cells and then in vivo in 4T1 mouse models.

## Results

### Synthesis and characterizations of CsI(Na) nanoparticles

**Fig. 1:**
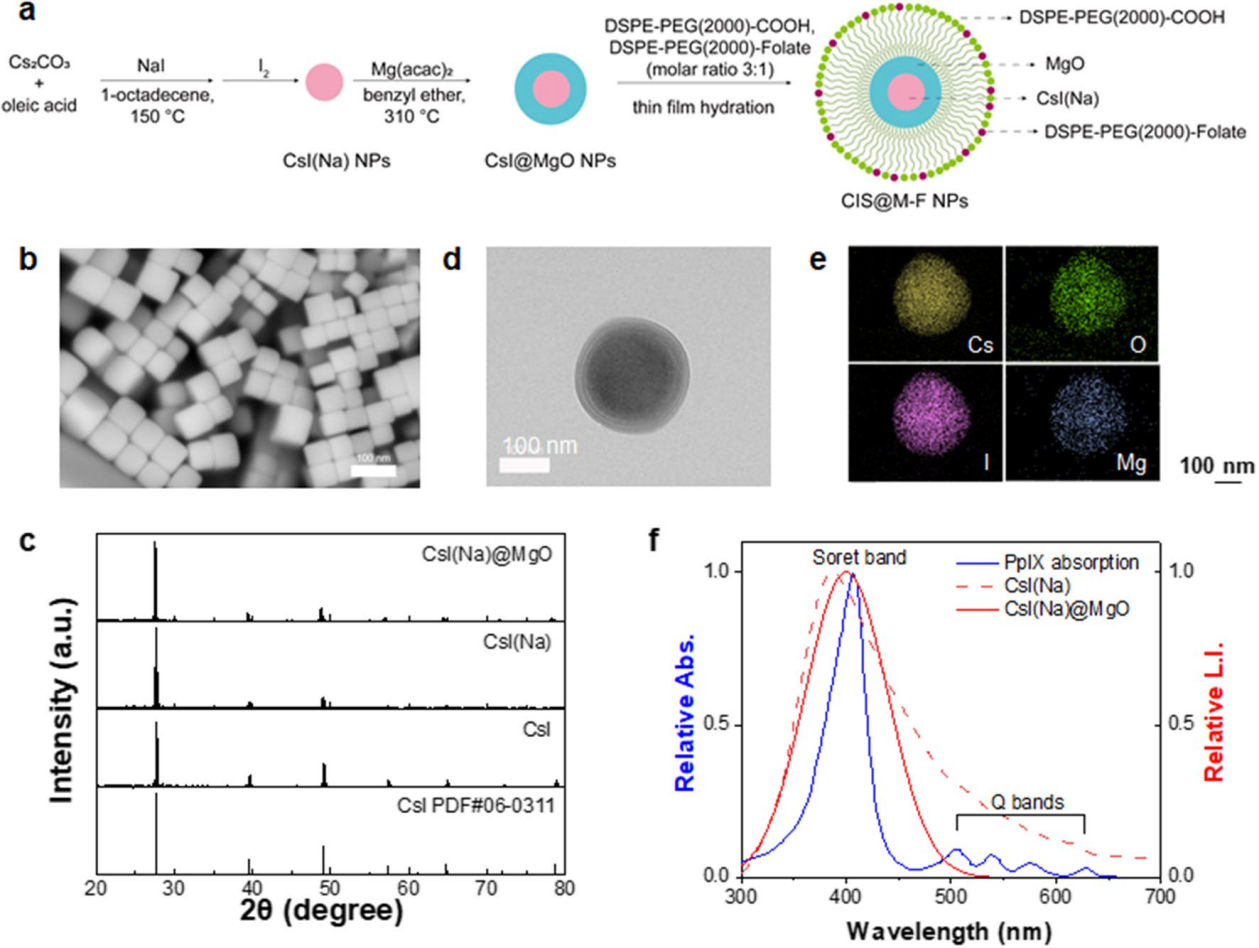
Synthesis and characterizations of CsI(Na) and CsI(Na)@MgO nanoparticles. **a** Schematic illustration of CsI(Na)@MgO synthesis and surface modification. **b** SEM of CsI(Na) nanoparticles. Scale bar, 100 nm. The average nanoparticle size was 55 ± 15 nm, determined by ImageJ. **c** XRD spectra of CsI(Na)@MgO, CsI(Na), and CsI (no Na dopant) nanoparticles, as well as a CsI standard (PDF#06-0311 from the JCPDS database). **d** TEM image of a single CsI(Na)@MgO nanoparticle. **e** EDX elemental analysis of a single CsI(Na)@MgO nanoparticle. Scale bar, 100 nm. **f** X-ray luminescence spectra of CsI(Na) (dashed red) and CsI(Na)@MgO (solid red) nanoparticles, along with the absorption spectrum of PpIX (blue). *Abs.* absorbance, *L.I.* luminescence intensity

We dissolved oleic acid and cesium carbonate in 1-octadecene and heated the solution to 150 °C (Fig. 1a). We then added oleylamine, 1,2-hexadecanediol, and NaI into the mixture, followed by the addition of I_2_. After reaction, we collected the products by centrifugation and redispersed them in hexane. The yield is ~ 90%. As-synthesized CsI(Na) nanoparticles were cubic (Fig. 1b) with an average size of 55.2 ± 15.1 nm. Energy-dispersive X-ray spectroscopy (EDX) confirmed that the Cs-I molar ratio was ~ 1:1 (Additional file 1: Fig. S1a). Inductively coupled plasma mass spectrometry (ICP-MS) found that the Na dopant was ~ 1%. Selected area electron diffraction (SAED) revealed a diffraction pattern that matches bulk CsI (Additional file 1: Fig. S1b) [43]. X-ray diffraction (XRD) also confirmed that the nanoparticles were CsI in composition and belonged to the Pm3m space group (No. 221, JCPDS#06-0311, Fig. 1c).

CsI(Na) is hygroscopic. To prevent fast degradation, we imparted a layer of MgO onto CsI(Na) nanoparticles via seed-mediated growth [44]. We chose MgO because it is low-toxic, stable at neutral pH, and biodegradable [45–49]. The resulting CsI(Na)@MgO nanoparticles were spherical and possessed a ~ 25.0-nm-thick shell (Fig. 1d, e, and Additional file 1: Fig. S1c). EDX confirmed the presence of Mg in the resulting nanoparticles, and that the Cs-I ratio remained at ~ 1:1 (Additional file 1: Fig. S1d). XRD identified characteristic CsI peaks but no MgO peaks (Fig. 1c), indicating that the coating is amorphous. We also studied the X-ray luminescence of CsI(Na)@MgO nanoparticles. Both CsI(Na) and CsI(Na)@MgO nanoparticles displayed an intense luminescence peak at ~ 410 nm (Fig. 1f), which agrees with the bulk material [50]. The luminescence peak overlapped well with the Soret band of PpIX (Fig. 1f), suggesting the potential for activating the PS with luminescence from CsI(Na).

## Evaluate RDT with CIS@M-F and 5-ALA in solutions

**Fig. 2:**
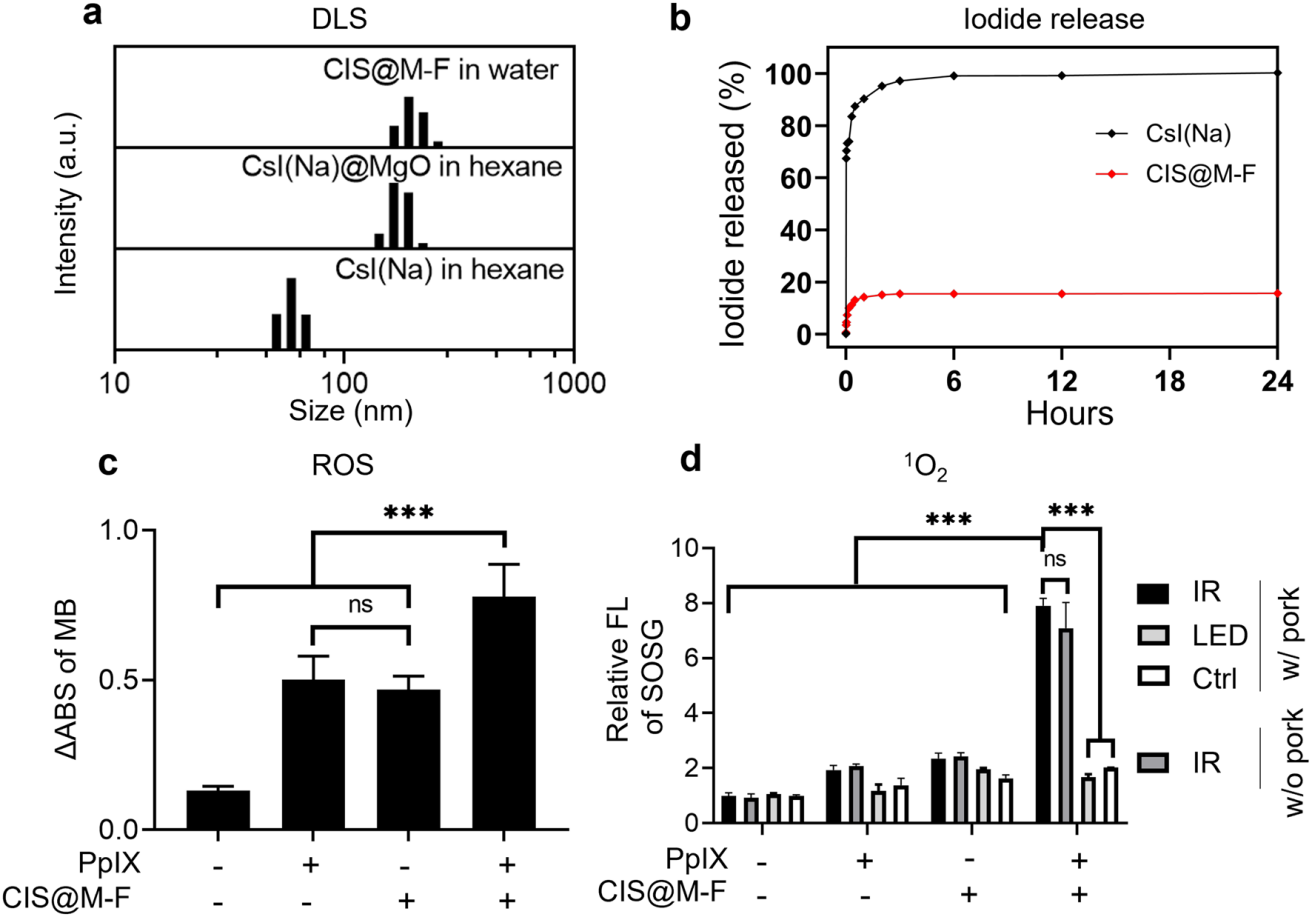
Stability and X-ray luminescence of CsI(Na)@MgO@DSPE-Folate nanoparticles (CIS@M-F). **a** DLS analysis of CsI(Na), CsI(Na)@MgO, and CIS@M-F nanoparticles. The hydrodynamic sizes are 58.7 ± 8.0 nm and 175.2 ± 37.5 nm, respectively, for CsI(Na) and CsI(Na)@MgO nanoparticles (in hexane). CIS@M-F can be stably dispersed in water, with a hydrodynamic size of 190.0 ± 30.0 nm. **b** Iodide release from CsI(Na) and CIS@M-F nanoparticles in PBS (pH 7.4). **c** ROS production under ionizing irradiation (IR, 5 Gy), measured with solutions containing CIS@M-F with or without PpIX, using methylene blue (MB) as an indicator. A decreased absorbance at 664 nm suggests an elevated ROS level. The experiments were repeated in quintuplicates. ***p < 0.001; ns, *p* > 0.05. **d** ^1^O_2_ generation under ionizing irradiation (IR, 5 Gy), measured with solutions containing CIS@M-F with or without PpIX, using SOSG (ex/em: 504/525 nm) as an indicator. Solutions were placed under 3-cm-thick pork. For comparison, LED light instead of X-rays was applied. The experiments were repeated in triplicates. ***p < 0.001; ns, *p* > 0.05

We coated CsI(Na)@MgO nanoparticles with DSPE-PEG-COOH and DSPE-PEG-Folate (molar ratio 3:1) through thin-film hydration. The resulting CsI(Na)@MgO@DSPE-Folate nanoparticles, hereafter referred to as CIS@M-F, are readily dispersed in aqueous solutions. Dynamic light scattering (DLS) showed that the average hydrodynamic size of CIS@M-F was 190.2 ± 30.1 nm (Fig. 2a). For comparison, CsI(Na)@MgO nanoparticles without a phospholipid coating were 175.2 ± 37.5 nm (in hexane, Fig. 2a). Z-potential analysis found that CIS@M-F were slightly negatively charged (− 22.5 mV, Additional file 1: Fig. S2a), which is attributed to the surface carboxyl groups.

We studied the degradation of CIS@M-F in PBS, tracing iodide released from the nanoparticles by ICP-MS. For comparison, we also tested uncoated CsI(Na) nanoparticles (which can be temporally dispersed in PBS). Uncoated CsI(Na) nanoparticles rapidly degraded, releasing > 90% of their iodine within 30 min (Fig. 2b). CIS@M-F showed significantly improved water stability. While a small amount of iodide was released at the beginning of incubation, virtually no iodide was leaked after 1 h. After 24 h, CIS@M-F remained a stable colloidal solution, while CsI(Na) solutions turned completely transparent due to degradation (Additional file 1: Fig. S2b, c).

To test whether CIS@M-F can activate PpIX under X-ray irradiation, we prepared solutions containing CIS@M-F, PpIX, and methylene blue (MB), and irradiated the solutions by X-rays (5 Gy). We observed a significant drop of 664 nm absorbance (Fig. 2c), suggesting MB being quenched by newly generated ROS. As a comparison, solutions containing CIS@M-F or PpIX only showed moderate MB quenching under the same condition. We next examined ^1^O_2_ production using singlet oxygen sensor green (SOSG) as a fluorogenic sensor. SOSG intensity was increased by more than 4-fold in solutions containing both CIS@M-F and PpIX (Fig. 2d), suggesting PpIX activation. As a comparison, solutions containing CIS@M-F or PpIX alone showed a minor SOSG intensity increase when irradiated. To validate that PpIX can be activated through RDT, we placed solutions containing CIS@M-F and PpIX under 3-cm-thick tissues and irradiated from atop (Additional file 1: Fig. S2d). We observed comparable levels of SOSG fluorescence upon X-ray radiation (Fig. 2d). On the contrary, LED light failed to activate PpIX under this condition. Overall, our solution studies support that RDT can be activated under irradiation if both PpIX and CIS@M-F are present.

### Evaluate RDT in vitro in cells treated with CIS@M-F and 5-ALA

**Fig. 3:**
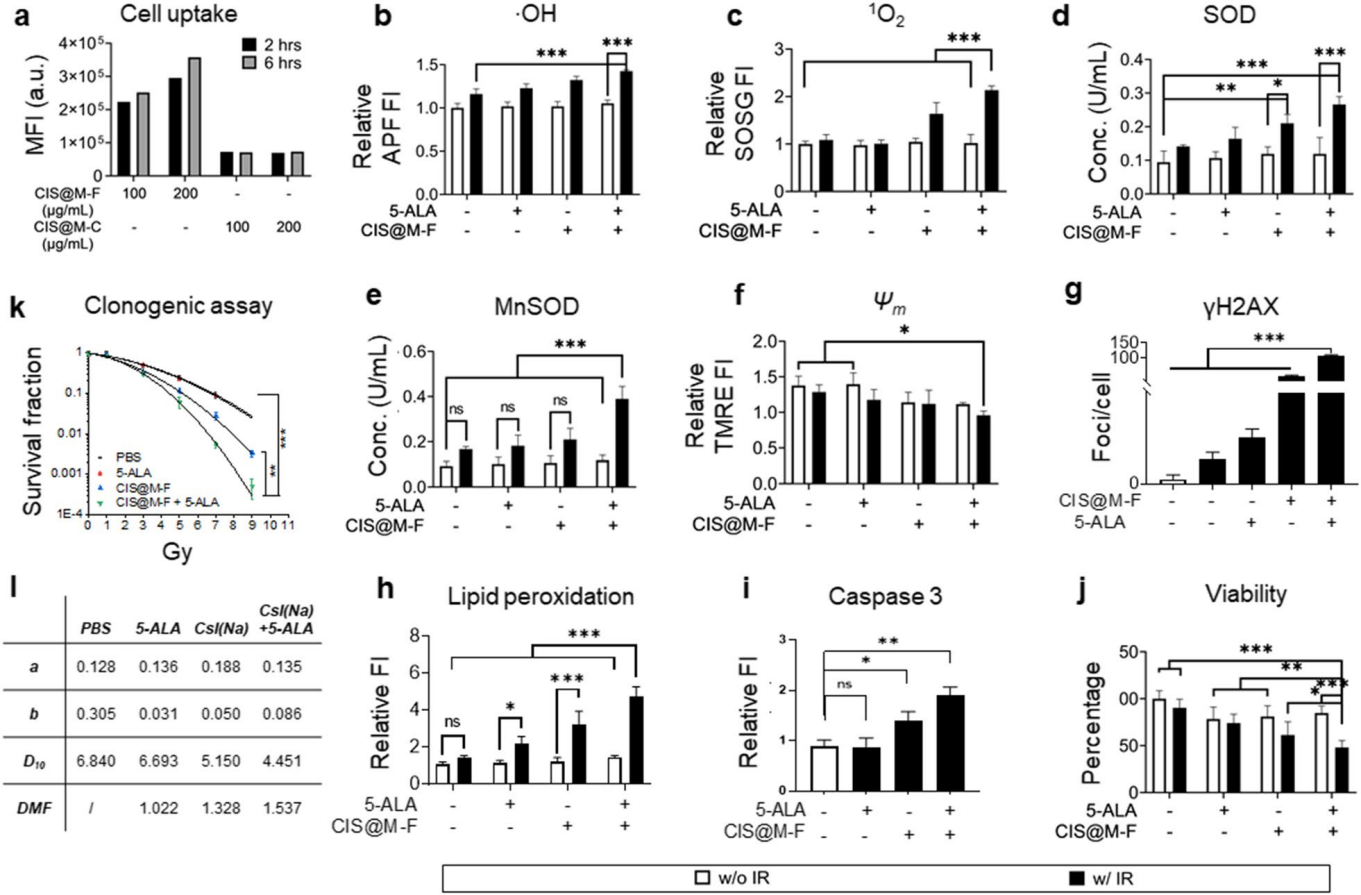
In vitro RDT with CIS@M-F and 5-ALA, evaluated with 4T1 cells. **a** Cell uptake, investigated with Rhodamine B labeled CIS@M-F or CIS@M-C (CsI(Na)@MgO nanoparticles coated with DSPE-PEG-COOH only) nanoparticles using flow cytometry. MFI, median fluorescence intensity. Increased cell uptake was observed with CIS@M-F compared to CIS@M-C at both 100 and 200 µg/mL. **b**–**e** Impact of CIS@M-F and 5-ALA on cellular oxidative stress. 4T1 cells were incubated with CIS@M-F (100 µg mL^−1^), 5-ALA (200 µg mL^−1^), or their combination, followed by IR (5 Gy). All experiments were repeated in quintuplicates. **b** Cellular hydroxyl radical levels, measured with APF (ex/em: 490/515 nm). **c** Cellular ^1^O_2_ levels, measured with SOSG (ex/em: 504/525 nm). Cytosol SOD (**d**) and mitochondrial MnSOD (**e**) activities, measured with Superoxide Dismutase Assay Kit. **f** Mitochondrial membrane potentials (*Ψ*_*m*_), measured with TMRE assay. **g** Double-strand DNA beaks, measured with anti-rH2AX staining. Positively stained foci per cells were quantified by ImageJ. **h** Lipid peroxidation, measured with C11-BIDOPY (ex/em: 488/510 nm) assay. **i** Cell viability, measured with ATP bioluminescence assay at 24 h. **j** Tumorigenicity, measured with clonogenic assay at a range of radiation doses (0–9 Gy; n = 3). **k** Summary of linear-quadratic (*S* = *e*^−(*a*D+*b*D^2)^) fitting results, based on clonogenic assay results from **j**. *D*_10_, dose required to achieve 10% survival. DMF, does modifying factor, based on *D*_10_ values. *p < 0.05; **p < 0.01; ***p < 0.001; ns, *p* > 0.05

We first studied CIS@M-F uptake by 4T1 cells, in which folate receptor is upregulated. To this end, we labeled CIS@M-F with rhodamine-B. For comparison, we also prepared rhodamine-B-labeled CsI(Na)@MgO nanoparticles coated with DSPE-PEG-COOH only (referred to as CIS@M-C). Flow cytometry found significantly increased median fluorescence intensity (MFI) of rhodamine-B in cells treated with CIS@M-F relative to those treated with CIS@M-C (Fig. 3a). Extending incubation time from 2 to 6 h increased uptake but the change was not marked (Fig. 3a). These results indicate that CIS@M-F were quickly internalized by cancer cells through receptor-mediated endocytosis.

We then investigated the impacts of RDT on cells. Briefly, 4T1 cells were incubated with CIS@M-F (100 µg mL^−1^) and 5-ALA (200 µg mL^−1^) for 3 h, and then treated with ionizing radiation (IR, 320 kV, 5 Gy). We chose this drug-radiation interval because the 5-ALA-to-PpIX conversion peaked at ~ 3 h (Additional file 1: Fig. S3a, b). Note that CIS@M-F and 5-ALA are not toxic to cells at the above therapeutic doses when there is no radiation (Additional file 1: Fig. S3c, d).

We first examined intracellular ROS level changes. Relative to un-irradiated cells, cells treated with IR alone showed an increased level of hydroxyl radical, measured with aminophenyl fluorescein or APF, a fluorogenic sensor of ·OH (Fig. 3b); this is mainly attributed to radiolysis of water. Treatment with 5-ALA prior to IR slightly elevated ·OH relative to IR alone but the increase was insignificant (*p* = 0.3865). Pre-treatment with CIS@M-F or a combination of CIS@M-F and 5-ALA moderately increased ·OH levels. The increase is attributed to high-Z element effects, which was observed with other nanoparticles [51]. Meanwhile, SOSG fluorescence intensity was not increased in cells treated with IR alone but more than doubled when cells were pre-treated with the CIS@M-F and 5-ALA combination (Fig. 3c). The result is consistent with observations made from solutions, supporting that RDT is activated in cells pre-treated with CIS@M-F and 5-ALA prior to IR.

Next, we evaluated the activities of superoxide dismutase (SOD) and manganese-dependent superoxide dismutase (MnSOD) in cells treated with CIS@M-F, 5-ALA, or their combination, with or without radiation. We observed significantly increased SOD and MnSOD activities in cells treated with CIS@M-F + 5-ALA + IR (Fig. 3d, e), indicating cell response to elevated ROS. In particular, MnSOD activity was more than 3 times higher than cells treated with CIS@M-F + 5-ALA or IR alone (Fig. 3e), which is attributed to the fact that PpIX activation in focused on mitochondria. Mitochondria-targeted activation was supported by the observation that mitochondria membrane potential (Δ*Ψ*_*m*_) significantly dropped in cells treated with CIS@M-F + 5-ALA + IR (Fig. 3f). Note that destructed mitochondria may promote secondary ROS that further oxidative stress [52].

Elevated ROS may cause oxidative damage to a broad range of biomolecules such as DNA, lipids, and proteins. γH2AX staining revealed a significant increase of positively stained foci per cell in cells treated with CIS@M-F + 5-ALA + IR relative to IR alone (Fig. 3 g and Additional file 1: Fig. S3e). Consistent with APF results, CIS@M-F + IR moderately increased double-strand breaks which is attributed to nanoparticle high-Z effects (Fig. 3g). C11-BODIPY staining found a significant increase of 510-nm fluorescence in cells treated with CIS@M-F + 5-ALA + IR (Fig. 3h), suggesting elevated lipid peroxidation. Extensive oxidative damages, including damage to the mitochondria, triggered apoptosis, which was evidenced with increased caspase-3 activity (Fig. 3i). ATP bioluminescence viability assay also confirmed reduced cell viability when cells were treated with the CIS@M-F + 5-ALA + IR (Fig. 3j).

Lastly, we evaluated how RDT affects cancer cell clonogenicity. We treated 4T1 cells with the same amounts of CIS@M-F (100 µg mL^−1^) and 5-ALA (200 µg mL^−1^) as the other in vitro experiments but varied the radiation doses (0–9 Gy). After 14 days, the survival fraction (*S*) relative to un-treated controls was calculated and fit into a linear-quadratic equation, *S* = *e*^−(*a*D+*b*D^2)^, where *D* is the radiation dose and *a* and *b* are fitting coefficients (Fig. 3k and Figure S4a). CIS@M-F + 5-ALA + IR reduced the number of colonies formed at all radiation doses. *D*_10_, dose required to achieve 10% clonogenic survival, was 4.45 (Fig. 3i and Additional file 1: Fig. S4b). This corresponds to a dose modifying factor (DMF) of 1.54. As a comparison, *D*_10_ values were 5.15 and 6.69 for CIS@M-F + IR and 5-ALA + IR, respectively, corresponding to DMFs of 1.33 and 1.02, respectively (Fig. 3i). Overall, our in vitro results support that under irradiation, the CIS@M-F-and-5-ALA combination enables RDT, promoting reduction in cell proliferation and reproduction relative to IR alone.

### Evaluate RDT with CIS@M-F and 5-ALA in vivo

**Fig. 4:**
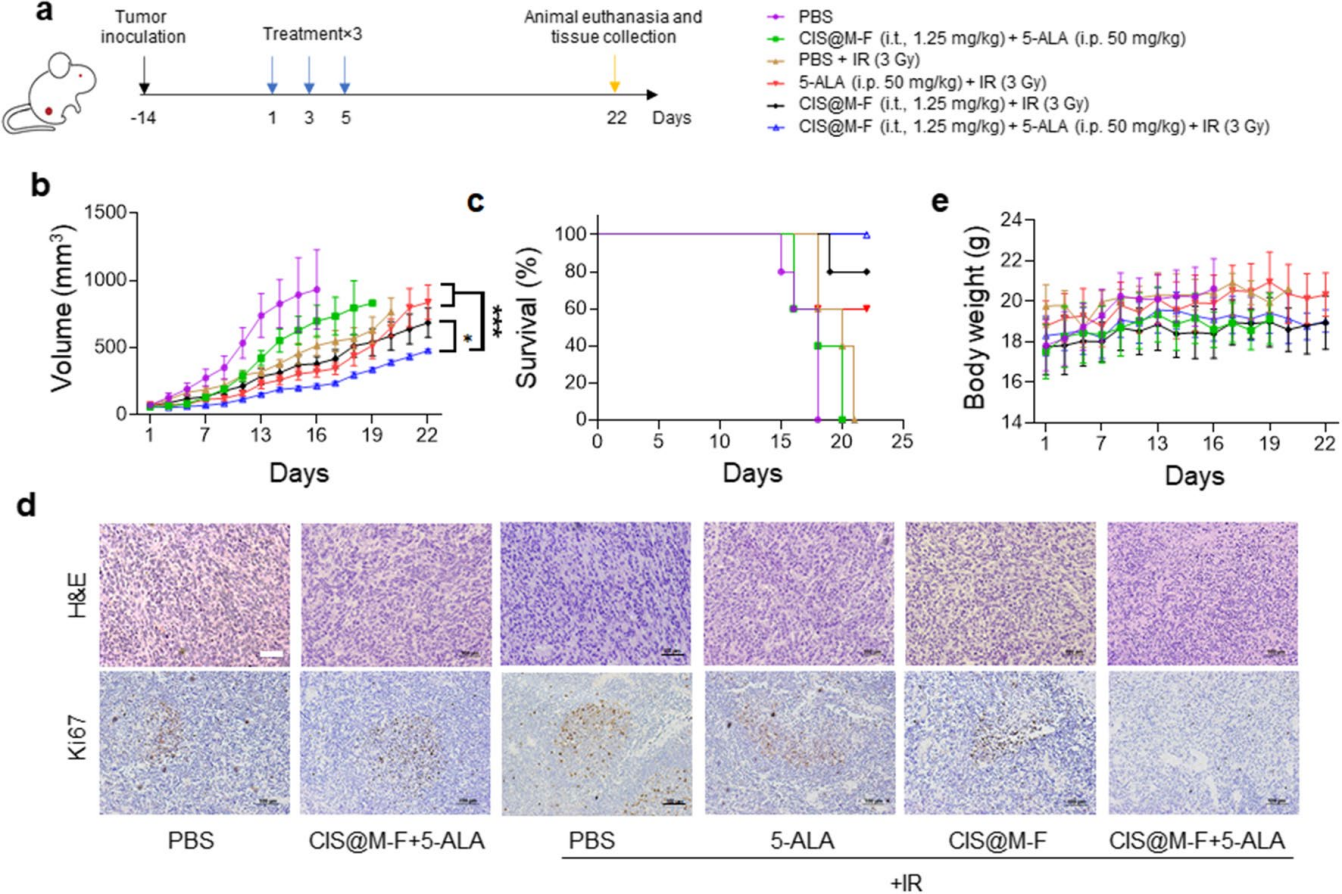
In vivo studies to evaluate the efficacy of RDT with CIS@M-F and 5-ALA. Experiments were performed in 4T1-tumor bearing balb/c mice. **a** Scheme of experimental design. On Day 1, animals received one of the following regimens, including PBS plus ionizing radiation (PBS + IR), 5-ALA with IR (5-ALA + IR), CIS@M-F plus IR (CIS@M-F + IR), PBS only (PBS), CIS@M-F plus 5-ALA without IR (CIS@M-F + 5-ALA), and CIS@M-F plus 5-ALA plus irradiation (CIS@M-F + 5-ALA + IR). 5-ALA (50 mg kg^−1^ in PBS) was i.p. administered, while CIS@M-F in PBS (1.25 mg kg^−1^) were intratumorally administered 2 h after the 5-ALA injection. IR (3 Gy) was applied to tumors 1 h after CIS@M-F administration. Two more sessions of treatment were given on Days 3 and 5. Animals were euthanized after 3 weeks or when a humane endpoint was reached. **b** Tumor growth curves. *p < 0.05; ***p < 0.001. **c** Kaplan Meier survival curves. **d** Post-mortem staining of tumor tissues, with H&E (upper) and Ki67 (lower). Scale bar, 100 μm. **e** Animal body weight curves

**Fig. 5:**
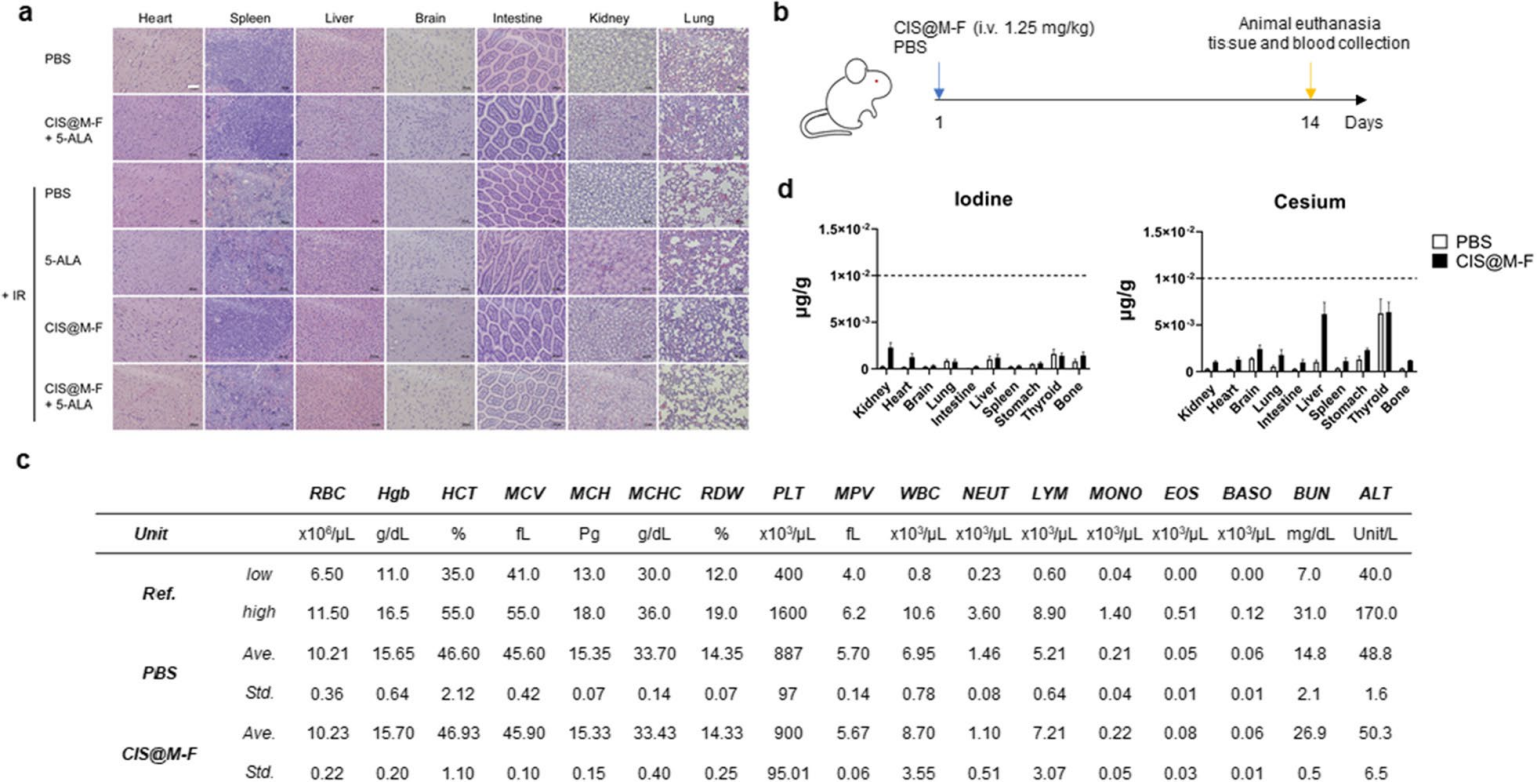
Safety and clearance of CIS@M-F. **a** H&E staining of tissues harvested from animals treated with regimens detailed in Fig. 4. Scale bar, 100 μm. **b** Scheme of experimental plans. In a separate experiment, CIS@M-F (1.25 mg/kg) or carrier only (PBS) was i.v. administered into healthy balb/c mice (n = 5). Animals were euthanized after 2 weeks for CBC and serum biochem analyses. In addition, remaining cesium and iodine in tissues were analyzed by ICP-MS. **c** Summary of CBC and serum biochem results, along with reported normal ranges of the indices. *RBC* red blood cells, *Hgb* hemoglobin, *HCT* hematocrit, *MCV* mean corpuscular volume, *MCH* mean corpuscular hemoglobin, *MCHC* mean corpuscular hemoglobin concentration, *RDW* red cell distribution width, *PLT* platelets, *MPV* mean platelet volume, *WBC* white blood cells, *NEUT* neutrophils, *LYM* lymphocytes, *MONO* monocytes, *EOS* eosinophils, *BASO* basophils, *BUN* blood urea nitrogen, *ALT* alanine transaminase. **d** Iodine (left) and cesium (right) remaining in organ tissues on Day 14. Contents of both elements fell well below 10 ng/g

We inoculated 4T1 cells into the right flanks of 5-6-week female nude mice. When tumor volume reached 50 mm^3^, we intraperitoneally (i.p.) administered 50 mg kg^−1^ 5-ALA (Day 1). The same or a similar dose is commonly used in small animal studies and induces PpIX accumulation in tumors after 3 h [34, 53, 54]. We intratumorally (i.t.) injected CIS@M-F (1.25 mg kg^−1^) after 1 h and delivered 3 Gy of radiation to the tumor area after 3 h (CIS@M-F + 5-ALA + IR, n = 5). The rest of the animal body was protected with lead. Two more treatment sessions were applied on Days 3 and 5 (Fig. 4a). Control treatments included PBS only, CIS@M-F + 5-ALA, CIS@M-F + IR, 5-ALA + IR, and IR alone (n = 5). Without treatment, 40% of the animals reached a humane endpoint within 16 days (Fig. 4a, b). IR alone was moderately therapeutic, inhibiting tumor growth by 26.2% on Day 16 (Fig. 4a). Animals in the 5-ALA + IR and CIS@M-F + IR groups exhibited tumor inhibition rates at 45.5% and 53.9%, respectively; the differences however were insignificant relative to IR alone (*p* = 0.5363 and 0.8056, respectively). By contrast, animals in the CIS@M-F + 5-ALA + IR group led to a tumor inhibition of 164.3% compared to IR alone (Fig. 4b, p = 0.0019). All mice in the CIS@M-F + 5-ALA + IR group remained alive after three weeks (Fig. 4c). Histopathology found reduced cell density and decreased levels of positive-Ki67 staining in tumors from the CIS@M-F + 5-ALA + IR group (Fig. 4d). Meanwhile, there was no acute toxicity nor body weight drops throughout the experiment (Fig. 4e). H&E staining found no signs of toxicity to normal tissues (Fig. 5a).

To better understand the side effects and clearance of CIS@M-F, in a separate study, we intravenously injected CIS@M-F into healthy balb/c mice and collected blood and tissues on Day 14 for complete blood counts (CBCs), serum biochemistry, and histopathology analyses (Fig. 5b). All CBC indices and liver and kidney function markers including alanine transaminase (ALT) and urea nitrogen (BUN) were within normal ranges [55] (Fig. 5c). H&E staining found no abnormalities in all tissues (Additional file 1: Fig. S5). Post-mortem ICP-MS studies revealed that the Cs and I levels in all tissues fell to baseline or close-to-baseline levels after 2 weeks (Fig. 5d). Overall, our in vivo studies confirm that RDT by CIS@M-F + 5-ALA can significantly enhance tumor suppression without causing additional toxicities.

## Conclusions

While CsI(Na) is an established scintillator material, CsI(Na) nanoparticles have seldom been explored. Employing CsI(Na) nanoparticles for RDT has not been reported. Herein we successfully synthesized CsI(Na) nanoparticles and, for water protection, coated them with MgO. We showed that X-ray luminescence from CsI(Na) nanoparticles can activate PpIX in 5-ALA-treated cells, enhancing mitochondria, DNA, and lipid damages thereby sensitizing cancer cells to RT. This strategy is distinct from conventional RDT approaches in that the scintillator (CIS@M-F) and PS (5-ALA) are administered separately rather than as a conjugate. CsI(Na)@MgO nanoparticles are synthesized by scalable wet chemistry. The approach is less affected by issues such as batch-to-batch variations, complex nanostructures, and suboptimal control in PS loading and release. Other benefits include high tumor selectivity (PpIX selectively accumulates in cancer cells and its activation takes place only when both CsI(Na) NPs and X-rays are present) and high efficiency (the PDT component focuses on mitochondria, which are sensitive to phototoxicities). Some have reported on the radio-sensitizing effects of 5-ALA [56–58]. In our in vitro and in vivo studies, however, we did not observe therapeutic benefits when 5-ALA was applied alone. This might be attributed to differences in radiation doses and animal models. In future studies, we will optimize the nanoparticle size, crystallinity, and dopant amount for improving the light output of the nanoparticles. It is possible to change the dopant from sodium to other metals, which would shift the luminescence; in accompany with the change, other photosensitizers with matching excitation wavelengths may be used. It is also worth exploring the impact of coating on the nanoparticles’ stability, luminescence intensity, and luminescence duration.

It is worth pointing out that RDT is practically a radiosensitizing strategy [18]. Its role is improving the efficacy of radiotherapy rather than eradicating tumors as a stand-alone treatment. A high radiation dose would certainly lead to improved tumor suppression but may obscure the benefits of radiosensitization as radiation alone could be effective. For proof-of-concept, we applied 9 Gy over three sessions, which is normal in small animal research. In future studies, we are certainly interested in exploring the benefits of our RDT at higher total radiation doses or different fraction doses. In the current investigation, we intratumorally injected CsI(Na) nanoparticles in therapy studies, which is common among RDT studies [22, 26, 27, 59]. The injection route is viable for treating cancer types such as breast, prostate, and skin cancers [22, 31, 60]. It is possible to systemically administer CsI(Na) nanoparticles, whereby they accumulate in tumors through passive or active targeting. Tumor-targeting ligands other than folic acid may be imparted to the nanoparticle surface depending on targeted indications.

As afore-mentioned, one major advantage of the current approach is the low toxicity of both the PS and the scintillator. 5-ALA is FDA-approved and has been used in clinical PDT and image-guided brain surgery. CsI(Na) nanoparticles degrade into alkali and halogen ions that are safely excreted after treatment. The demonstrated biocompatibility and potential scalability of the nanoplatform ideally position this technology for further developments and clinical translation.

## Methods

### Materials

Cs_2_CO_3_ (99%, Sigma, Cat#441902), I_2_ (≥ 99.8%, Sigma, Cat#207772), NaI (≥ 99.5%, Sigma, Cat#383112), magnesium acetylacetonate dihydrate, (Mg(acac)_2,_ 98%, Sigma, Cat#129577), 1-Octadecene (C_18_H_36_, technical grade, 90%, Sigma, Cat#0806), oleic acid (C_18_H_34_O_2_, technical grade, 90%, Sigma, Cat#364525), oleylamine (C_18_H_35_NH_2_, technical grade, 70%, Sigma, Cat#07805), 1,2-tetradecanediol (technical grade, 90%, Sigma, Cat#260290), 1,2-hexadecanediol (technical grade, 90%, Sigma, Cat#213748), hexane (C_6_H_14_, ≥ 99%, Sigma, Cat#139386), ethanol (anhydrous, Sigma, Cat#443611), benzyl ether (98%, Sigma, Cat#108014), chloroform (CHCl_3_, ≥ 99.8%, Fisher Scientific), 1,2-dipalmitoyl-sn-glycero-3-phosphoethanolamine-*N*-(lissamine rhodamine B sulfonyl) (ammonium salt) (16:0 Liss Rhod PE) (Avanti, Cat#810,158), 1,2-distearoyl-sn-glycero-3-phosphoethanolamine-*N*-[carboxy(polyethylene glycol)-2000] (sodium salt) (DSPE-PEG(2000) Carboxylic Acid) (Avanti, Cat#880135), 1,2-distearoyl-sn-glycero-3-phosphoethanolamine-*N*-[folate(polyethylene glycol)-2000] (ammonium salt) DSPE-PEG(2000) Folate (Avanti, Cat#880124), methylene blue (C_16_H_18_ClN_3_S·xH_2_O, powder, ≥ 82%, Sigma), phosphate buffer saline (PBS, pH 7.2), Milli-Q Water (H_2_O, 18.2 MΩ.cm@25 °C).

### Synthesis of CsI(Na) nanoparticles

0.203 g Cs_2_CO_3_ was mixed with 10 mL 1-octadecene and 1 mL oleic acid in a 100 mL flask. The solution was heated to 150 °C and maintained at this temperature for 30 min with magnetic stirring. Next, 1 mL oleylamine, 0.02954 g 1,2-hexadecanediol, and 0.01 g NaI were added into the mixture. After reacting for 10 min, the solution was cooled to room temperature, and 0.3165 g I_2_ were added. The flask was then sealed and stirred for another 3 h. CsI nanoparticles were collected by centrifugation and washed with a 1:1 hexane and ethanol mixture 3 times.

### Synthesis of CsI(Na)@MgO nanoparticles

In a typical reaction, 20 mg CsI(Na) nanoparticles in 5 mL hexane was mixed with 0.02 g Mg(acac)_2_, 0.053 g 1,2-tetradecanediol, and 20 mL benzyl ether in a 100 mL three-neck flask. The solution was heated to 120 °C under Argon protection and maintained at this temperature for 20 min. The solution was further heated to 310 °C before the addition of 1 mL pre-heated oleic acid. The reaction continued for 10 min before being cooled to room temperature. The product was collected by centrifugation and washed with a 1:1 hexane and ethanol mixture 3 times.

### Synthesis of CIS@M-F

20 mg CsI(Na)@MgO nanoparticles were dispersed in 2 mL chloroform. Into the solution, 75 µL of DSPE-PEG(2000)-COOH in chloroform (10 mg mL^−1^) and 25 µL DSPE-PEG(2000)-Folate in chloroform (10 mg mL^−1^) were added. The mixture was stirred at room temperature overnight, and the solvent was removed by rotary evaporation. The nanoparticles were dispersed in PBS and passed through a desalting column before use.

### Synthesis of CIS@M-F or CIS@M-C with rhodamine B labelling

20 mg CsI(Na)@MgO nanoparticles were dispersed in 2 mL chloroform. Into the solution, 50 µL of DSPE-PEG(2000)-COOH in chloroform (10 mg mL^−1^) and 25 µL DSPE-PEG(2000)-Folate in chloroform (10 mg mL^−1^) together with 25 µL of 16:0 Liss Rhod PE in chloroform (10 mg mL^−1^) were added. The mixture was stirred at room temperature overnight, and the solvent was removed by rotary evaporation. The nanoparticles were dispersed in PBS and passed through a desalting column before use.

### Nanoparticle characterizations

Nanoparticle crystallinity was assessed using the Bruker D8-Advance X-ray diffraction (XRD) diffractometer with Cu Kα radiation (λ = 1.5418 Å) at a scanning rate of 10° min^−1^. The hydrodynamic sizes and surface charges of the particles were characterized on a Malvern Zetasizer Nano ZS system. Nanoparticle size, morphology, and elemental analysis was characterized using a Scanning Electron Microscope (FE-SEM Thermo Fisher Teneo) which was equipped with an EDX system and Transmission Electron Microscope (FEI Tecnai20 and FEI Tecnai G2 F30 Hi-Res TEM). Nanoparticle composition was analyzed by Inductively Coupled Plasma Atomic Emission Spectroscopy using an Xseries II ICP/MS system (Thermo Electron Corporation). An iodide-selective electrode was used to conduct release experiments in PBS solutions of nanoparticles at room temperature (Mettler Toledo perfectION™).

### Radical production

100 µL PBS control, 100 µg mL^−1^ CIS@M-F, 0.04 M PpIX, and 100 µg mL^−1^ CIS@M-F plus 0.04 M PpIX solutions were prepared, distributed into a 96-well plate, and irradiated with 5 Gy X-ray (X-RAD 320). 80 µL 1 µм methylene blue was added to each well immediately following radiation, and the plate was shaken and kept in darkness at room temperature for 5 min before testing. A UV–vis spectrometer was then used to record absorbances (664 nm).

### Cell culturing

4T1 breast cancer cells were used for in vitro and in vivo studies. Cells were grown in RPMI1640 medium which was supplemented with 10% FBS and 100 units mL^−1^ of penicillin (ATCC). Cells were maintained in a humidified, 5% carbon dioxide (CO_2_) atmosphere at 37 °C.

### ATP viability assay to test nanoparticle and 5-ALA toxicity

The ATP viability assay was performed according to the manufacturer’s protocol (PerkinElmer, ATPlite 1step Luminescence Assay Cat#6016736)[61]. 4T1 cells were seeded at 5000 cells/well in a white 96-well plate. After 24 h of incubation, CIS@M-F and 5-ALA (18.75, 37.5, 75, 150, 300, 600 µg mL^-1^ for CIS@M-F;19.5, 78.1, 312.5, 1250 µg mL^−1^ for 5-ALA) were added to each well for another 24 h of incubation. The ATP kit solution was then added to each well, and a 96-well microplate reader was used to measure total luminescence. Cell viability was calculated as a percentage of the luminescence of the untreated control.

### ATP viability assay to test X-PDT efficacy

The ATP viability assay was performed according to the manufacturer’s protocol (PerkinElmer, ATPlite 1step Luminescence Assay Cat#6016736). 4T1 cells were seeded at 5000 cells/well in a white 96-well plate and incubated for 24 h before the addition of 20 µg 5-ALA to each well. Cells were incubated for 1 h before the addition of 10 µg CIS@M-F. Cells were then incubated for 2 h before being irradiated (5 Gy). The plate was returned to the incubator and maintained in darkness for 24 h before the ATP luminescence test.

### Intracellular PpIX analysis

PpIX extraction was performed according to a published protocol [62]. Cells were seeded in a 96-well plate at 5000 cells/well. After 24 h of incubation, cells were trypsinized, harvested by centrifugation, and redispersed in 5% HCl at 37 °C for an hour. Following incubation in acid, the supernatant was collected and fluorescence signals (ex/em: 406/604 nm) were recorded.

### APF assay

ROS (reactive oxygen species) were measured with the APF assay (Invitrogen^™^ Cat#A36003) [63]. 4T1 cells were seeded in 96-well plates at 5000 cells/well. After 24 h, nanoparticles in 100 µL RPMI medium or medium only were added to each well and incubated for 2 h before being irradiated (5 Gy by X-Rad 320). Thereafter, the plate was incubated with 100 µL APF solution (2 µм) for 30 min at room temperature in the dark. Lastly, the medium was diluted with an equal volume of fresh PBS, and fluorescence signals (ex/em: 490/515 nm) were analyzed on a microplate reader (Biotek).

### SOSG (singlet oxygen) assay

The SOSG assay was conducted following the vendor’s protocol (Invitrogen^™^ Cat#S36002) [64]. 4T1 cells were seeded in 96-well plates at 5000 cells/well. Following 24 h of incubation, the medium was aspirated and 20 µL SOSG solution (5 µм) with 100 µg mL^−1^ CIS@M-F in 100 µL medium were added to each well. After 1 h, 20 µL 5-ALA (1 mg mL^−1^) was added. 5 Gy radiation was delivered following another 2 h of incubation. Fluorescence signals (ex/em: 504/525 nm) were recorded on a microplate reader (Biotek).

### Cell uptake studies

Cell uptake of CIS@M-C and CIS@M-F nanoparticles were analyzed on a CytoFLEX flow cytometer. 4T1 cells were seeded at 0.5 × 10^6^ cells/well into 6-well plates. Then, nanoparticles were incubated with cells at a final nanoparticle concentration of 100 µg mL^−1^. Both CIS@M-F and CIS@M-C were labeled with Rhodamine B following a published protocol [65]. Following either 2 or 6 h of incubation, cells were harvested for flow cytometer analysis, and the MFI was recorded.

### SOD activity

SOD activity was assessed following the vendor’s protocol (Cayman Chemical Cat#706002). 4T1 cells were seeded into 6-well plates at 1 million cells/well. After 24 h, 2 mL RPMI medium containing 100 µg mL^−1^ CIS@M-F and 200 mL^−1^ 5-ALA were added to each well. 5 Gy X-ray irradiation was delivered after 4 h. Immediately following irradiation cells were washed with PBS three times and collected with a rubber scraper. Cell pellets were subjected to differential centrifugation at 4 °C and 12,000 rpm for 20 min to separate the mitochondrial and cytosolic fractions. Both the supernatant and mitochondrion were collected, aliquoted, sonicated and transferred into a 96-well plate. Test kit solution was added to each well, and the 96-well plate was shaken for 10 min in the dark at room temperature before measurement. Absorbance (450 nm) was measured on a microplate reader (Biotek).

### Lipid peroxidation

The Image-iT Lipid Peroxidation Kit (Invitrogen^™^ Cat#C10445) was used to assess lipid peroxidation. 4T1 cells were seeded into 96-well plates at 5000 cells/well. Following 24 h of incubation, 200 µg mL^−1^ 5-ALA or 100 µg mL^−1^ CIS@M-F were added to each well. Following 4 h of incubation, the plate was irradiated (5 Gy). The Image-iT Lipid Peroxidation dye was added to each well, and the plate was incubated at 37 °C and 5% CO_2_ for 30 min. Green (ex/em: 488/510 nm) fluorescence intensity was used to quantitate lipid peroxidation.

### Caspase-3 activity

4T1 cells were incubated with CIS@M-F (100 µg mL^−1^) and 5-ALA (200 µg mL^−1^) for 2 h prior to receiving 5 Gy X-ray irradiation. Control treatments included CIS@M-F, 5-ALA, or PBS. Following 24 h of incubation, cells were stained with the FAM-FLICA^®^ Caspase-3/7 kit (Immunochemistry, Cat#94) following the manufacturer’s protocol. The caspase-3 activity was evaluated by measuring fluorescence signals (ex/em: 488/530 nm) on a microplate reader (Synergy Mx, BioTeK).

### Mitochondrial membrane potential (ΔΨ_m_)

Mitochondrial potential was assessed using the TMRE staining kit following the vendor’s protocol (Abcam Cat#ab113852). 4T1 cells were incubated with CIS@M-F (100 µg mL^−1^) for 2 h followed by 5-ALA (200 µg mL^−1^) for 3 h before receiving 5 Gy irradiation. Control treatments included CIS@M-F, 5-ALA, or PBS with or without irradiation. The medium was aspirated after 24 h, and cells were incubated in TMRE staining solution for 15 min. Fluorescence signals (ex/em: 549/575 nm) were measured on a microplate reader.

### γH2AX

DNA damage was evaluated using anti-rH2AX (Alexa 647 labeled) antibodies (Millipore Sigma, Cat# 07-164-AF647). Briefly, 4T1 cells were seeded onto a 4-well imaging chamber at a density of 10,000 cells/well and incubated for 24 h. After washing, the cells were incubated with CIS@M-F (100 µg mL^−1^) for 2 h followed by 5-ALA (200 µg mL^−1^) for 3 h before receiving 5 Gy irradiation. Control treatments included CIS@M-F, 5-ALA, or PBS with or without irradiation. After 1 h, cells were collected, fixed, permeabilized, and stained with anti-rH2AX antibodies following the vendor’s protocol. Fluorescent images were acquired on a Zeiss LSM 710 confocal microscope. ImageJ was used to count the number of foci per cell.

### Clonogenic assay

Clonogenic assays were performed using a modified protocol [66]. 4T1 cells were pre-seeded into 6-well plates. After 24 h, cells were incubated with CIS@M-F (100 µg mL^−1^) for 2 h followed by 5-ALA (200 µg mL^−1^) for 3 h before receiving 0, 1, 3, 5, 7, and 9 Gy of radiation. Treated cells were trypsinized, replanted in petri dishes (100 * 15 mm), and incubated at 37 °C with 5% CO_2_. After 14 days, cells were rinsed carefully with PBS, fixed in 2–3 mL of 6.0% glutaraldehyde solutions, and treated with 1 mL of 0.5% crystal violet. After 10 min, cells were rinsed with D.I. water and dried before colony counting. Colonies containing at least 50 stained cells were included in survival fraction (SF) calculations.

### In vivo therapy studies

Animal studies were performed according to a protocol (A2020 06-004-R1) approved by the Institutional Animal Care and Use Committee (IACUC) of the University of Georgia. The animals were maintained under pathogen-free conditions. 4T1 tumors were established by subcutaneously injecting 2 × 10^5^ cells in 50 µL PBS into the right flanks of 5–6-week old female BALB/c mice (Charles River). When tumor volume reached 50 mm^3^, the animals were randomly divided into 6 groups (n = 5) and received the following treatments (Day 1): PBS plus ionizing radiation (PBS + IR), 5-ALA with irradiation (5-ALA + IR), CIS@M-F plus irradiation (CIS@M-F + IR), PBS only (PBS), CIS@M-F plus 5-ALA, no irradiation (CIS@M-F + 5-ALA), or CIS@M-F plus 5-ALA plus irradiation (CIS@M-F + 5-ALA + IR). 5-ALA (50 mg kg^−1^ in PBS) was administered intraperitoneally. CIS@M-F in PBS (1.25 mg kg^−1^) were intratumorally injected 2 h after the 5-ALA injection. A 320 KV cabinet irradiator (X-RAD 320, Precision X-ray, Inc.) was used to irradiate (3 Gy) tumors 1 h after CIS@M-F administration, while the rest of the animal body was protected with lead. Animals underwent two additional treatment sessions on Days 3 and 5. Tumor size and body weight were inspected every 3 days. Tumor dimensions were measured with a caliper. Tumor volume was estimated by calculating (length) × (width)^2^/2. Animals were euthanized after 22 days. Tumors were dissected and sliced for H&E and Ki67 staining. Organs including the heart, spleen, liver, brain, intestine, kidney, and lung were also harvested for H&E staining.

### Biodistribution studies

5–6 week-old female BALB/c mice (Charles River) were intravenously injected with CIS@M-F (50 µL, 1.25 mg kg^−1^) or PBS (control) *via* the tail vein. All mice were sacrificed after 2 weeks. Blood was collected through cardiac puncture for complete blood count (CBC), BUN, and ALT measurements. Major organs, including the heart, spleen, liver, brain, intestine, kidney, and lung were harvested. Half of the tissues were weighted, homogenized, and digested in hot nitric acid. Supernatants were subjected to ICP-MS analysis to measure tissue concentrations of cesium and iodine in tissues (µg/g of tissue). The remaining tissues were fixed and sliced for H&E staining.

### Statistical analysis

All quantitative data were shown as mean ± SD. Statistical analysis was conducted using student’s *t* or ANOVA test. *p < 0.05, **p < 0.01, ***p < 0.001.

## Supplementary Information

**Additional file 1.**  It includes methods, EDX and SAED of CsI(Na) nanoparticles, SEM and EDX of of CsI(Na)@MgO nanoparticles, zeta potential of CIS@M-F and CIS@M-C, in vitro studies investigating 5-ALA conversion to PpIX, cytotoxicity of 5-ALA and CIS@M-F in the absence of IR, rH2AX staining results, clonogenic assay results, H&E staining of organ tissues as well as serum BUN and ALT levels after animals being i.v. administrated with CIS@M-F.
