# Supplementary material for: Radiodynamic therapy with CsI(na)@MgO nanoparticles and 5-aminolevulinic acid
Source: J Nanobiotechnology. 2022 Jul 16;20:330. doi: 10.1186/s12951-022-01537-z (PMC9288050; doi:10.1186/s12951-022-01537-z)
Supplement: Supplementary file 1 — Additional file 1. It includes methods, EDX and SAED of CsI(Na) nanoparticles, SEM and EDX of of CsI(Na)@MgO nanoparticles, zeta potential of CIS@M-F and CIS@M-C, in vitro studies investigating 5-ALA conversion to PpIX, cytotoxicity of 5-ALA and CIS@M-F in the absence of IR, rH2AX staining results, clonogenic assay results, H&E staining of organ tissues as well as serum BUN and ALT levels after animals being i.v. administrated with CIS@M-F. [file 12951_2022_1537_MOESM1_ESM.pdf]

Additional file 1 for

Radiodynamic Therapy with CsI(Na)@MgO Nanoparticles and 5-Aminolevulinic Acid

Fangchao Jiang[1], Chaebin Lee[1], Weizhong Zhang[1], Wen Jiang[1], Zhengwei Cao[1], Harrison Byron Chong[1], Wei Yang[1], Shuyue Zhan[1], Jianwen Li[1], Yong Teng[2], Zibo Li[3,], Jin Xie[1,*]*

[1] Department of Chemistry, University of Georgia, Athens, GA 30602, USA
[2] Department of Hematology and Medical Oncology & Winship Cancer Institute, Emory University School of Medicine, Atlanta, GA, 30322, USA
[3] Department of Radiology, University of North Carolina at Chapel Hill, Chapel Hill, NC 27599, USA

*Corresponding author:
Zibo Li: zibo_li@med.unc.edu
Jin Xie: jinxie@uga.edu

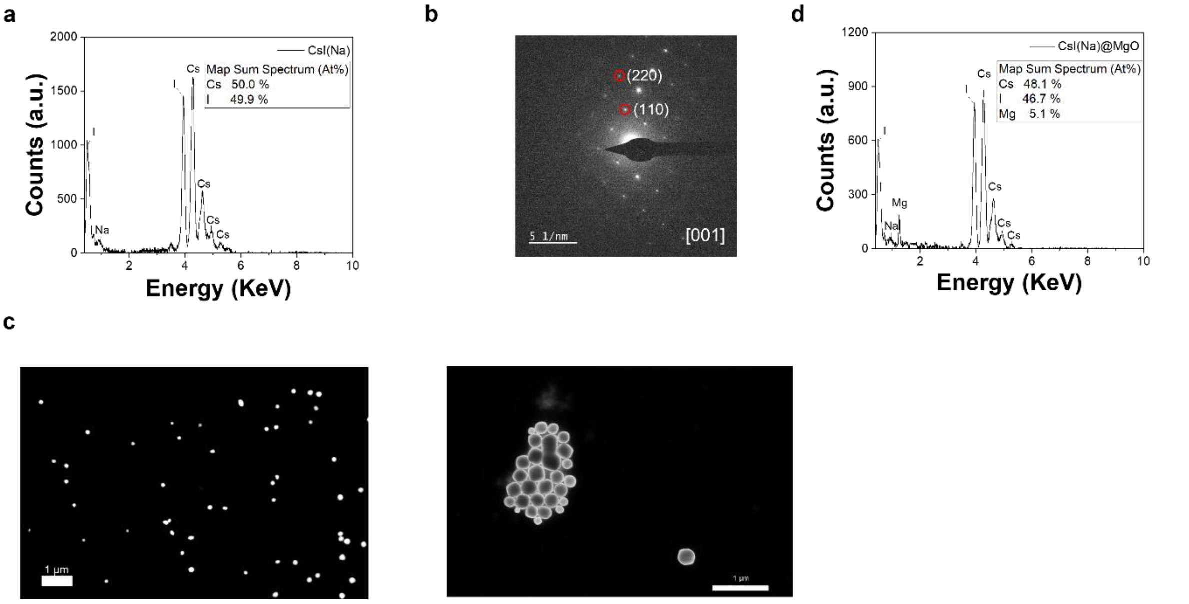

Figure S1. a) Elemental analysis of CsI(Na) nanoparticles by EDX. b) Selected area electron diffraction (SAED) of a single CsI(Na) nanoparticle under HRTEM. c) TEM images of CsI(Na)@MgO nanoparticles. Scale bars, 1 μm. d) EDX elemental analysis of CsI(Na)@MgO nanoparticles.

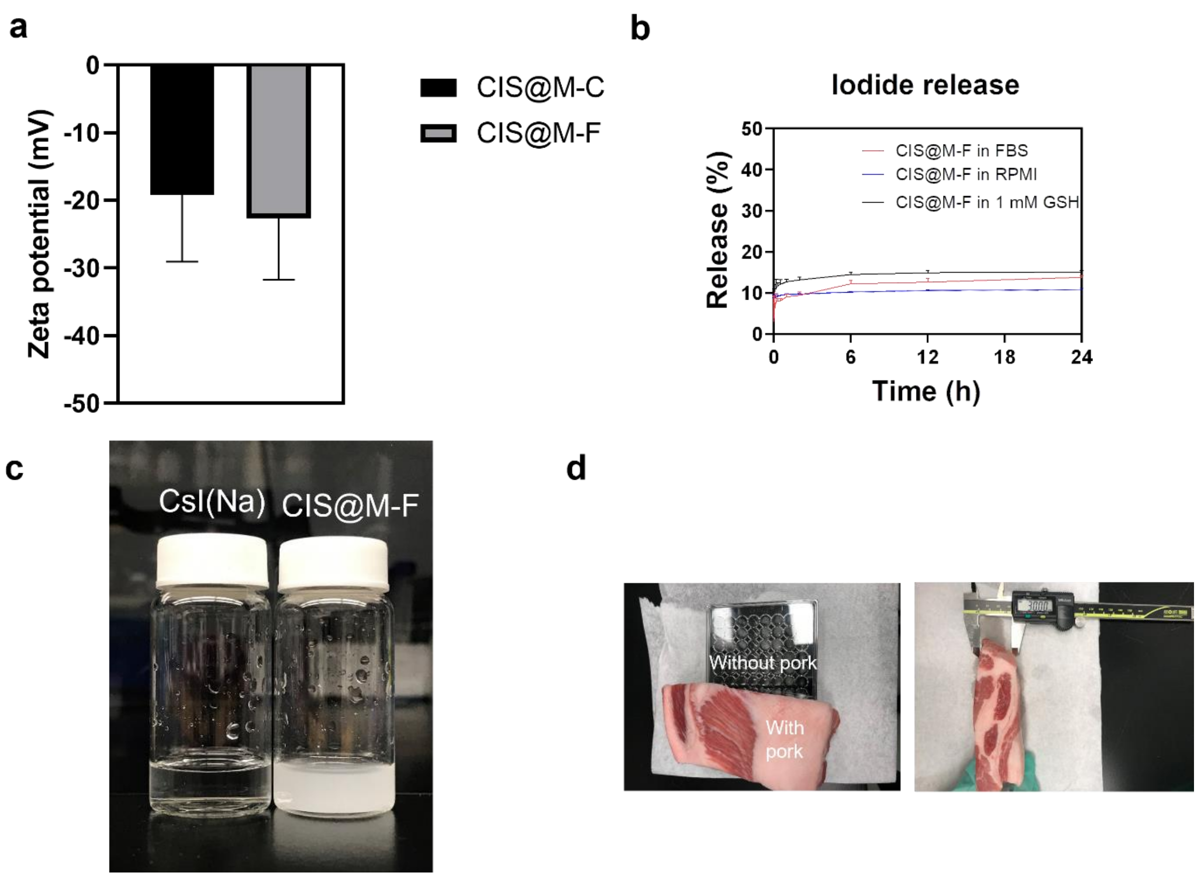

Figure S2. a) Z-potentials CIS@M-C and CIS@M-F nanoparticles, measured in D.I. water. b) Iodide release from CIS@M-F nanoparticles in FBS, RPMI cell culture medium, and D.I. water supplemented with 1 mM GSH. c) Photos of CsI(Na) and CIS@M-F nanoparticles, taken after 24 hours of incubation in water. d) Setup of experiment to evaluate X-PDT activation. Solutions containing CIS@M-F, PpIX, and SOSG was irradiated with X-rays or LED light, with or without 3-cm pork placed between solutions and radiation sources. Experimental results are detailed in Figure 2d.

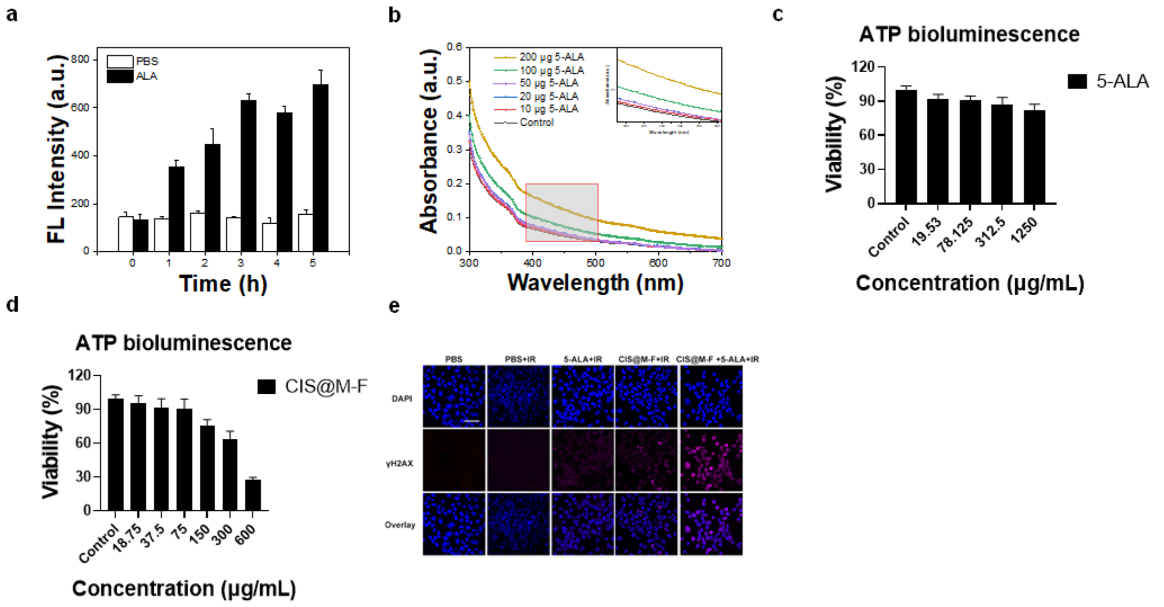

Figure S3. a) 5-ALA was converted to PpIX in cells, measured by measuring PpIX fluorescence. The signals were plateaued after ~ 3h. b) Characteristic PpIX absorbance was increased when cells were incubated with 5-ALA. The increase was 5-ALA-concentration dependent. c) Cell viability, measured with ATP bioluminescence assay at 24 h of incubation. 4T1 cells were incubated with 5-ALA up to 1250 μg/mL. d) Viability of 4T1 cells when incubated with CIS@M-F in the absence of ionizing radiation (IR), measured with ATP bioluminescence assay. e) Double-strand breaks, measured with rH2AX staining. 4T1 cells were treated with CIS@M-F+5-ALA+IR, CIS@M-F+IR, 5-ALA+IR, IR, and PBS. Positively stained foci per cells were quantified by ImageJ and the results were summarized in Figure 3g. Scale bar, 50 μm. Red, rH2AX; blue, DAPI.

a

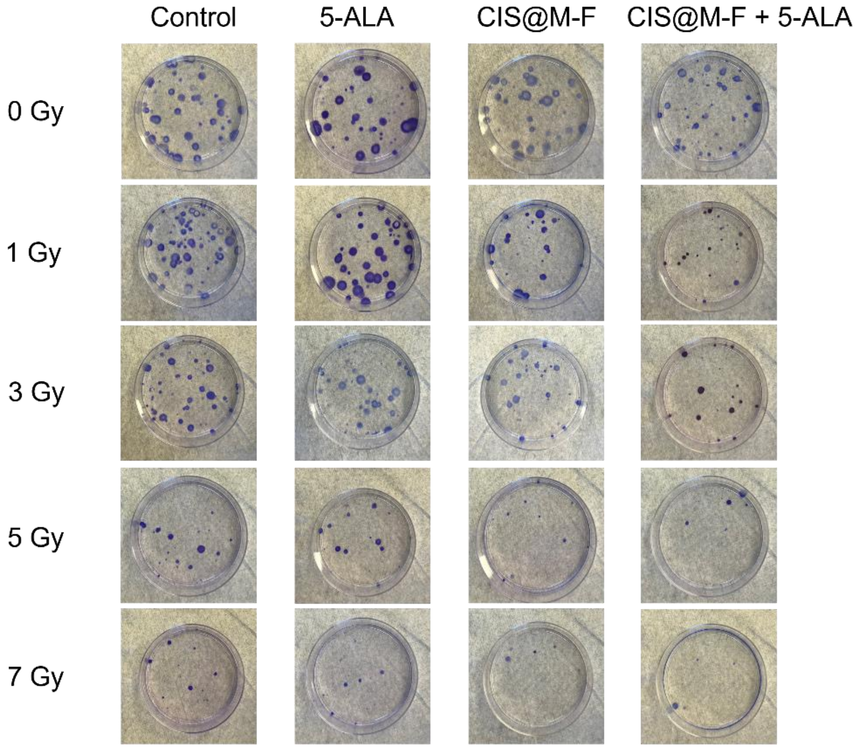

b

Summary of data fitting results

Group	*a*	*b*	*a/b*	*SF2*	*SF5*	D_{10}	DMR_{10}	*REF2*	*REF5*
PBS	0.128	0.0305	4.1967	0.6852	0.2460	6.8402	/	/	/
5-ALA	0.1359	0.0311	4.3698	0.6729	0.2329	6.6927	1.0220	1.0183	1.0562
CIS@M-F	0.1875	0.0504	3.7202	0.5618	0.1111	5.1503	1.3281	1.2197	2.2142
CIS@M-F+ 5-ALA	0.1354	0.0858	1.5781	0.5411	0.0595	4.4511	1.5367	1.2663	4.1345

Figure S4. a) Representative images of clonogenic assay results from different treatment groups. b) Summary of data fitting based on linear-quadratic equation. S = exp-(aD+bD^2), where S is the cell survival fraction, D is the radiation dose, and a&b are fitting coefficients. Radiation enhancement factors based on survival fraction relative to PBS (IR alone) at 2 Gy (REF2) and 5 Gy (REF5) as well as D_{10} (dose required to achieve 10% clonogenic survival) (DMF_{10}) were calculated and compared.

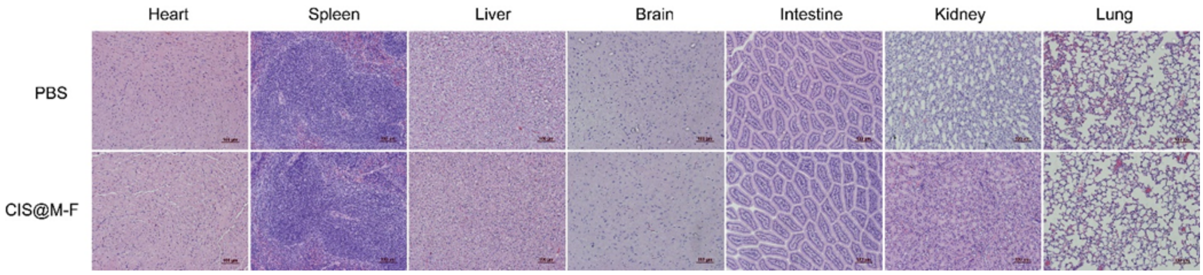

Figure S5. H&E staining of major organ tissues. Mice were i.v. injected with CIS@M-F (1.25 mg/kg) or carrier only (PBS) and euthanized after two weeks (n=5). Scale bars, 100 µm.
